# Power and Autistic Traits

**DOI:** 10.3389/fpsyg.2016.01290

**Published:** 2016-08-31

**Authors:** Geir Overskeid

**Affiliations:** Department of Psychology, University of OsloOslo, Norway

**Keywords:** autism, broader autism phenotype, power, sex differences, testosterone

## Abstract

Autistic traits can help people gain and sustain power, and has probably done so throughout history, says the present paper. A number of testable claims follow from this assumption. First, the powerful should have more autistic traits than others – which they do appear to have. Among other things, powerful people, and those with many autistic traits, tend to prefer solitary activities and are often aloof. Moreover, they are often rigid and socially insensitive, low on empathy and with low scores on the trait of agreeableness – and as a rule they do not have many friends. Both groups are also more self-centered than others, more honest, less submissive, more sensitive to slights, and with a stronger tendency to engage in abstract thinking. They tend to behave in bossy or dominant ways, and their moral judgment is more based on rules than on feelings. In addition to experimental evidence, I cite biographies showing that a surprising number of presidents, prime ministers and other powerful people seem to have had traits like those in question – and interestingly, in animals, leaders are often rigid and insensitive to group members’ needs and feelings, mostly acting the way they are themselves inclined to, not responding much to others. Problem solving is important in leadership, and people with many autistic traits appear often to be better thinkers than typical subjects with similar IQs. However, these and other congruities could be coincidences. Hence the question of whether traits the two groups have in common also have a common cause constitutes a strong test of the paper’s thesis – and a common cause does appear to exist, in the form of testosterone’s effects on the central nervous system. Finally, there is evidence that, other things equal, powerful men have more reproductive success than others. If men wielding power do indeed have more autistic traits than those less powerful, this will lead to, other things equal, such traits becoming more common – which can help explain the prevalence of autistic traits.

## Introduction

To understand human behavior, we must understand power – “a universal and indispensable feature of social organization,” in the words of Turner (2005, p. 1). Russell (1938, p. 4) calls power “the fundamental concept in social science.”

Some have more power than others, and powerful people tend to have certain characteristics, such as expertise (Stehr and Grundmann, 2011), resources (Galbraith, 1983), and male gender (Baumeister, 2010). Though interesting, this does not tell us why people with such features gain and hold on to power more often than others.

I shall argue that those who gain and sustain power often have many autistic traits, and that the autistic traits of these individuals have often helped them reach their powerful position. This hypothesis leads to several testable claims, some supported by the extant literature, and some in need of further investigation. If the hypothesis is correct, it can also help explain why men have more autistic traits than women, why there are more powerful men than women, and why autistic traits are so prevalent. This may strike some readers as counterintuitive. Hence I shall do my best to make clear why the hypothesis is not only testable, already having considerable support, but also why it seems the most parsimonious explanation of a large body of data from several areas of research.

## The Broader Autism Phenotype

Some people have autism or related conditions. The present paper is not primarily about them. But then there are those who have more autistic traits than the average person, while still not qualifying for a diagnosis. The latter category is often referred to as the “broader autism phenotype” (BAP; Ingersoll and Wainer, 2014). More people are assumed to belong in this category, thus having “subclinical” autistic traits, than those who have full-blown autism (see Baron-Cohen, 2008). Given that in the USA, one boy in 42 and one girl in 189 are now assumed to have an autism spectrum disorder (Baio, 2014), the subclinical group could be quite substantial.

In discussing relevant traits in the powerful, I sometimes refer to findings from autism research, and sometimes to what we know about the BAP. This seems reasonable, since there is no clear boundary between autism and the BAP, and, in the words of Ingersoll and Wainer (2014, p. 43): “… research indicates that the BAP is comprised of a number of characteristics seen in individuals with ASD (autism spectrum disorder), but to a lesser extent.”

Among other things, persons with subclinical autistic traits are often aloof, rigid, anxious, shy, suspicious, and self-conscious (see Ingersoll and Wainer, 2014). They also tend to be neurotic introverts who score low on the trait of agreeableness (Wainer et al., 2011), and as a rule they do not have many friends (Jobe and Williams White, 2007). Are these people who can win elections and gain power? Before you say no, let’s have a look at some very powerful people – not to give them a diagnosis, but to get a picture of their tendency, if any, toward autism.

## Eccentric and Powerful

John Adams, the second president of the United States, was a “stiffly formal and aloof” man (Ferling, 1992/2010, p. 170). He was called cold, pugnacious (DeGregorio, 2009, p. 19), stubborn (McCullough, 2001), and said to have a “ready temper” (DeGregorio, 2009, p. 19). He seemed rigid, in the sense that “[a]ll his life he adhered to the habits he had learned in his youth …” (Ferling, 1992/2010, p. 173). It also appears he had frequent fits of depression, described as “black despair” (DeGregorio, 2009, p. 19). Depression is common in autism (see Stewart et al., 2006).

“There are very few People in this World, with whom I can bear to converse,” wrote Adams (1776). He could discuss business with colleagues, “but I am never happy in their Company. This has made me a Recluse …,” he continued. Adams lacked the facility for making small talk, and “social gatherings were maddeningly disagreeable ordeals for him” (Ferling, 1992/2010, p. 170). Socializing and making small talk is difficult and uncomfortable for most people with autism (e.g., Baron-Cohen, 2008).

Adams also seems to have had a certain tendency toward intense interests in restricted areas, which is often seen in autism (e.g., Asperger, 1944). Not only did Adams spend “a lifetime in politics” (Ferling, 1992/2010, p. 452), he was also “if not obsessed with the law, at least extraordinarily committed to it…” Indeed, his legal studies were said to have “taken on a life of their own, spurring him to master obscure… precedents … far beyond the needs of his practice” (Ryerson, 2005, p. 90).

Let us move across the Atlantic, and to times much closer to the present. After 10 years as chancellor of the Exchequer, Gordon Brown was prime minister of the United Kingdom from 2007 to 2010. Called “aloof” (Davis, 2009) and claimed not to function well socially, he is said to be oblivious to the impacts of his actions on others (Seldon and Lodge, 2011). We saw above that those with many autistic traits are often aloof (Wainer et al., 2011). They also tend to be socially insensitive (Asperger, 1961).

Dubbed “humorless” (Richards, 2010, p. 315), and unable to make small talk (Freedland, 2007), Brown is also said to have problems getting across to others (Robinson, 2013), with an “apparent inability to show empathy with voters” (Brindle, 2008). Humor and empathy also tends to be problematic for those who have autism (Baron-Cohen, 2008).

Additionally, Brown may have some tendency toward intense and restricted interests. He has spent a lifetime in politics, even writing a doctoral thesis on political change in his native Scotland (Brown, 1981). A biography of the former prime minister has been called “a study in political obsession” (Sylvester, 2004). Yet Brown appears to have room for one more enthusiasm. His Labor Party homepage claims he was “football^1^-obsessed” as a little boy (The Labour Party, n.d.), and as prime minister he was still “totally obsessed” with the sport (Hencke, 2008), demonstrating “encyclopedic knowledge” of football and footballers (McBride, 2014, p. 30).

Brown is also called “obsessive” more generally (Harris, 2006) – indeed, a “control freak” (Harris, 2006), or even a “control freak’s control freak” (Rawnsley, 2010, p. 473), with an “inflexible style” (Theakston, 2011, p. 92) and a “neurotic attention to detail” (Richards, 2010, p. 382), all traits often found in autistic people (see Baron-Cohen, 2008). Lord Turnbull, former head of Britain’s civil service, has claimed that Brown operated with “Stalinist ruthlessness” (see Timmins, 2007). This seems not to be an untypical assessment (Theakston, 2011), and may indicate a lack of empathy.

Physical clumsiness is also often observed in people with autism (Papadopoulos et al., 2014). Harris (2006) describes Brown’s “physical awkwardness” and calls him “autistic.”

Harold Macmillan was prime minister of the United Kingdom from 1957 to 1963, and is generally seen as one of the more successful British heads of government (Historical Rankings of Prime Ministers of the United Kingdom, 2016). “A shy and sensitive child” (Matthew, 2011), Macmillan had a “lonely childhood” (Thorpe, 2010, p. 18), and was “not popular” at school (Thorpe, 2010, p. 24). Children with autism are often seen as shy (Jones and Frederickson, 2010) and sensitive (Markram and Markram, 2010). They tend to have a lonely childhood (Bauminger and Kasari, 2000) and to be unpopular at school (Asperger, 1961).

Macmillan grew up to be “a rather awkward figure” (Thorpe, 2010, p. 283) who called himself a “very buttoned-up person” (Horne, 1989, p. 12) – and people with autism have been called “awkward” since they were first described (Kanner, 1943, p. 229). “[F]riends … were, in truth, few and far between,” says Williams (2010, p. 473). Hence Macmillan could feel “deeply lonely” (Mount, 2011), and, said Thomas (1998, p. 180), “suffered from extremes of loneliness.”

Macmillan had a “habit of lecturing his colleagues” (Williams, 2010, p. 183), but an “inability to listen” (Mount, 2011). Williams (2010, p. 471) reports that even Queen Elizabeth was “bored at being lectured (by Macmillan) and not being listened to.” People with autism often monopolize conversations with lectures or monologs (see James, 2006).

A certain lack of empathy may be behind Macmillan’s reported “ability to ignore, often brutally, those who stood in his way” (see Williams, 2010, p. 174). “Again and again, one notices the callous insouciance …,” says Mount (2011).

At the same time, Macmillan is called “sensitive” (Thorpe, 2010, pp. 31, 105) “highly strung” (Horne, 1989, p. 13) and prone to recurrent depressions (Davidson, 2011), with a “lifelong hypochondria” (Matthew, 2011). At 37, he had what “seemed to have been a full-scale nervous breakdown” (Horne, 1989, p. 98), and “another bad collapse” at 49 (Mount, 2011). Motorically, he was described as “clumsy” (Van der Kloot, 2009, p. 4) with “a marked lack of prowess… for any kind of athletics” (Horne, 1989, p. 13).

The three people just described are one long dead American and two more recent characters. The two were Britons, however, from a nation said to appreciate eccentrics. Indeed, a political observer points out that many British heads of government have been somewhat eccentric, and feels the British electorate wants their prime ministers to be “slightly set apart, a little bit weird” (Hoggart, 2010). There is evidence that people with more than the average number of autistic traits are often seen as eccentric (see Ingersoll and Wainer, 2014). So before we continue, let’s have a look at just one more powerful person, one who wasn’t British.

Ronald Reagan was “aloof,” says Bolton (2008, p. 99). “Adored by so many, he was a man with no real friends,” says his biographer Morris (2004), who also calls the 40th president “remote,” “detached,” and often insensitive to others. But *system* appealed to him. Reagan “needed to impose order on chaos … he liked punctuality, symmetry, sureness” (Morris, 2004). A strong affinity for systemizing, another word for imposing order on chaos, is said to be a central feature of autism (Baron-Cohen et al., 2011). While Reagan’s diary is “remarkable for a near-total lack of interest in people as individuals” (Morris, 2004), the game of politics aroused an intense and restricted interest in the former actor. In his thirties he was already “so obsessed with trade unionism and anti-Communism as to lose interest in casual chat,” and his first wife divorced him citing politics as her co-respondent (see Morris, 2004).

## Characteristics of the Powerful

The biographical sketches above may indicate that it is not impossible for individuals described as cold, aloof, rigid, depressive, obsessive, socially insensitive, etc. to achieve power. More interesting, however, and a stronger test of the present hypothesis, are findings describing how those who feel powerful behave more generally.

It seems priming a feeling of power makes people more self-centered (Galinsky et al., 2006), or more focused on the self than are those who have less power (see Van Kleef et al., 2015).

Also, those feeling powerful seem to engage in more abstract thinking than do those who feel less powerful (Magee and Smith, 2013). People primed to feel powerful often seem more honest (Anderson and Berdahl, 2002), perhaps because power makes a person care less about what others might think (Galinsky et al., 2008). At the same time, those primed to feel powerful appear more sensitive to being slighted than subjects primed to feel they have less power (Sawaoka et al., 2015).

Powerful people also often appear suspicious about the causes of subordinates’ good behavior – not attributing it to selflessness or intrinsic motivation, but to their own power as managers to distribute reward and punishment (Kipnis, 1972). Simply priming the thought of being powerful appears to make subjects start thinking in similarly cynical ways (Inesi et al., 2012). It is relevant that agreeableness appears to correlate negatively with leadership success (Boudreau et al., 2001).

A feeling of power, it seems, also tends to reduce submissiveness. For instance, college students primed to feel powerful were less willing to try and reach goals their mothers had set for them than were members of a group who had engaged in a neutral priming procedure (Inesi and Rios, 2013). Gardner (1995) studied the biographies of people who were later to become very powerful leaders, and found that quite early in their lives, they were typically marked by “a willingness to confront individuals in authority” (Gardner, 1995, p. 286).

Compared to those who feel they lack power, those feeling powerful have a stronger preference for solitary activities (Conlon, 2012), and are more socially distant (Lammers et al., 2012), with an increased tendency to treat people as objects (Kipnis, 1972; Lammers and Stapel, 2011).

Based on biographical material, Ludwig (2002) studied a great number of individuals who had ruled a country in the 20th century, and found that “[o]ther than voicing their views on politics, they usually were not adept as conversationalists and did not feel comfortable in social settings…. Despite their outer veneer of sociability, they remained very private people …” (p. 336). When people who feel powerful do take part in conversations, they often monopolize them with lectures or monologs (Tost et al., 2013).

Furthermore, priming a feeling of power tends to give subjects more difficulty reading others’ emotions (Galinsky et al., 2006; Gonzaga et al., 2008), and more difficulty taking others’ perspective (Galinsky et al., 2006). Those feeling powerful also tend to have a strong and narrow focus on goals (see Hirsh et al., 2011), which may help explain the finding that this group seems *better* than controls at taking others’ perspectives when a concrete goal must be reached (Schmid Mast et al., 2009). The construct of perspective taking is related to empathy and empathic accuracy, which has been found to be reduced in subjects primed to feel powerful (van Kleef et al., 2008), as well as in real managers wielding structural power (Sherman et al., 2015).

Furthermore, as opposed to most people, there is evidence that the morality of those who feel powerful is based on rules, not affect (Lammers and Stapel, 2009). A feeling of power also frequently leaves people insensitive to social consequences (see Hirsh et al., 2011), and less easily influenced by others (Galinsky et al., 2008). It may also seem that very powerful people are lonely and depressed more often than others (Ludwig, 2002).

Luntz (2011) points out that many powerful business leaders are very detail-oriented. One such leader is James Sinegal, founder of Costco, the multi-billion retailer. Sinegal feels a focus on details is essential to success in his line of business, and says: “[S]how me a big-picture guy, and I’ll show you a guy who’s out of the picture” (see Luntz, 2011, p. 179). In Collins and Hansen’s (2011) study of business leaders selected for their unusual degree of success, these individuals were described as relentless truth-seekers not influenced much by others’ opinions, always seeking out hard data instead. Subjects primed to feel powerful are also more diligent, as long as they do not find a task uninteresting because it is “beneath” them (DeWall et al., 2011).

Finally, what is it people do when they exercise power? Ent et al. (2012, p. 620) states that “power is essentially the control over other people and over what happens to them.” Not surprisingly, a tendency to act dominantly predicts power attainment (see Anderson and Brion, 2014).

It seems, then, that certain ways of thinking and acting are typical of those who feel powerful. What may be most interesting to us, however, is that studies have often found the same tendencies and characteristics in those who have many autistic traits (see **Table 1**).

**Table 1:** Similarities among people high on testosterone, autistic traits, and power.

High testosterone	Autism or many autistic traits	Power
(1) Self-centered/strong focus on the self (Mehta et al., 2009^∗^; Wright et al., 2012^#^).	(1) Self-centered (Asperger, 1944). “… little doubt that classic autism involves a total focus on the self” (Baron-Cohen, 2005, p. 172).	(1) Self-centered/more focused on the self (Galinsky et al., 2006; Van Kleef et al., 2015).
(2) More honest (Wibral et al., 2012^#^).	(2) More honest (Baron-Cohen, 2007; Chevallier et al., 2012).	(2) More honest (Anderson and Berdahl, 2002).
(3) Sensitivity to slights (Eisenegger et al., 2010^#^; Yildirim and Derksen, 2012^†^).	(3) Sensitivity to slights (Pomeroy, 1998; Tantam, 2000).	(3) Sensitivity to slights (Sawaoka et al., 2015).
(4) More abstract thinking (in female subjects only, Amani et al., 2012^∗^).	(4) More abstract thinking (Asperger, 1944; Hayashi et al., 2008; Stevenson and Gernsbacher, 2013).	(4) More abstract thinking (Magee and Smith, 2013).
(5) Low submissiveness (Turan et al., 2014^∗^, but see Moskowitz et al., 2015^§^).	(5) Low submissiveness (Asperger, 1944; Attwood, 2007).	(5) Low submissiveness (Inesi and Rios, 2013).
(6) Lower trust (Bos et al., 2010^#^; De Neys et al., 2013^§^; Carré et al., 2014^∗^).	(6) High suspiciousness (Ingersoll and Wainer, 2014).	(6) Lower trust (Schilke et al., 2015). Increased suspiciousness (Kipnis, 1972).
(7) More forward manner in conversations (Dabbs et al., 2001^∗^).	(7) Often dominating the conversation (Asperger, 1961; Sevin et al., 2007).	(7) Likely to attempt to dominate conversations (Tost et al., 2013).
(8) Increased dominance (Nelson et al., 2012^§^; van Honk et al., 2014^†^, but see, e.g., van der Meij et al., 2012^§^; Moskowitz et al., 2015^§^).	(8) Bossy, controlling behavior (Baron-Cohen, 2008, but see De Pauw et al., 2011).	(8) Dominant behavior in general (Anderson and Brion, 2014).
(9) Preference for solitary activities/socially distant/more loneliness (Pennebaker et al., 2004^#^; Turan et al., 2014^∗^).	(9) Preference for solitary activities/socially distant (Kanner, 1943; Baron-Cohen, 2008). More loneliness (Bauminger and Kasari, 2000; Müller et al., 2008 – but see Chamberlain et al., 2007).	(9) Preference for solitary activities/socially distant (Conlon, 2012; Lammers et al., 2012). More loneliness (Ludwig, 2002, but see Waytz et al., 2015).
(10) Moral judgment more based on utilitarian rules (Carney and Mason, 2010^∗^; Montoya et al., 2013^#§^).	(10) Moral judgment more based on utilitarian rules (Gleichgerrcht et al., 2013).	(10) More rule-based moral thinking (Lammers and Stapel, 2009).
(11) Less influenced by the opinions of others (Wright et al., 2012^#^).	(11) Less influenced by the opinions of others (Asperger, 1961; Yafai et al., 2014; but see Bowler and Worley, 1994).	(11) Less influenced by the opinions of others (Galinsky et al., 2008; Collins and Hansen, 2011).
(12) More persistent (Welker and Carré, 2015^∗^).	(12) Gillberg (2002, p. 66) writes: “Many with Asperger syndrome… tend to give up easily. Many others are extremely persistent. Others still combine impatience in some settings… with extreme tenacity and persistence in other situations …”	(12) More persistent (Willis and Guinote, 2011).
(13) Reduced empathy and reduced recognition of emotions (Chapman et al., 2006^§^; van Honk et al., 2011^#§^; Auyeung et al., 2013^†^).	(13) Reduced empathy and reduced recognition of emotions (Baron-Cohen, 2011).	(13) Reduced empathy and reduced recognition of emotions (van Kleef et al., 2008; Sherman et al., 2015, but see Schmid Mast et al., 2009).
(14) More restricted interests (Knickmeyer et al., 2005^§^).	(14) More restricted interests (American Psychiatric Association, 2013).	(14) The greatest leaders have narrow interests (Ludwig, 2002). Narrow goal-focused attention (Hirsh et al., 2011).


*Regarding testosterone (T) references, the following symbols are used: An asterisk (^∗^)indicates that naturally occurring T was measured. A number sign (^#^) indicates that T was administered to subjects. A section sign (^§^) indicates that putative signs of prenatal T level were measured. A dagger (^†^) indicates that the source is a literature review. This table is meant to demonstrate parallel findings in three separate literatures, but does not represent a complete literature review.*

## Are They Really Autistic Traits?

The thesis of the present paper is that autistic traits can help people gain and sustain power. But traits found in one group of people can also be found in another. How do we know that what we discuss is related to autism? To the extent that powerful people have something in common in the way they differ from others, how do we know that they aren’t psychopaths, for example? It has been argued that psychopathic people can often be found high up in many organizations (e.g., Babiak and Hare, 2006; Boddy et al., 2010). And yes, as might be expected of psychopaths, the leaders discussed in the present paper appear at times to have lacked empathy. However, they do not seem to have much of the glib and superficial charm often ascribed to psychopaths, nor of other traits included in a much-used instrument like Hare’s (2003) Psychopathy Checklist, like pathological lying, parasitic lifestyle, and irresponsibility.

It’s been argued that many leaders are narcissistic (Maccoby, 2007), and, as pointed out by a reviewer, it must be hard for a leader to avoid becoming at least somewhat narcissistic, once he or she gains power. It we turn to diagnoses, however, a category such as “narcissistic personality disorder” in the DSM-5 (American Psychiatric Association, 2013) is probably not the right one for the powerful people discussed here. The lack of empathy can be there, but not typically the expectation to be recognized as superior without commensurate achievements, the preoccupation with fantasies of unlimited success, power, or ideal love, nor the belief that one should only associate with other special or high-status persons, to take the first three on the DSM-5 list of nine diagnostic criteria.

Could we instead be talking about schizoid, or perhaps obsessive-compulsive personality traits? People with such characteristics have much in common with those who have autism, to the extent that there is evidence of appreciable overlap among these categories (e.g., Constantino et al., 2009; Lugnegård et al., 2012). Quite a few characteristics, in other words, can reasonably be called autistic, as well as schizoid or obsessive-compulsive.

At the same time, some traits are more common in autistic people than in other personality types. For instance, people with schizoid personality disorder are often indifferent to either praise or criticism, they rarely appear angry, and take pleasure in few, if any activities (see Millon et al., 2004).

People with autism are typically sensitive to criticism (e.g., Fitzgerald, 2004). There are no indications that powerful people are typically indifferent to praise or criticism, and many descriptions of powerful people who are not. They also seem to want praise at least as much as others.

One example is Lyndon Johnson, who, it seems, was always seeking approval (Shesol, 1997). In presidents Washington and Nixon, it appears that both were the case. The first president “was overly sensitive to criticism and suffered from a lifelong need for approval,” says Chernow (2010, p. 11), his biographer. Richard Nixon, too, it seems, sought constant approval (Frank, 2013; Stanley, 2013), while also reacting to criticism with strong negative emotion (Karpowitz, 2009). Ludwig (2002) gives more examples of powerful people who yearn for approval and are sensitive to criticism.

Approval or praise can also serve as potent reinforcers of autistic patients’ behavior (e.g., Walton and Ingersoll, 2013). Both powerful and autistic people will often get irritated easily (e.g., Bower, 2007; Baron-Cohen, 2008), and both groups tend to be strongly engaged in one or a few interests, like politics or trains – and sometimes both. British prime minister Harold Wilson was fascinated by trains and railway timetables (Jenkins, 2004).

Roy Jenkins, former president of the European Commission, described as a boy as “obsessively numerate… addicted to numbers” (Campbell, 2014), said he and Wilson had common interests, not only is statistical details, but also in “railway timetables and railway stations” (Hennessy, 2001, p. 363). Campbell (2014) recounts that all his life Jenkins loved making lists and grading things, and says his love of numbers was what brought him to trainspotting. As a boy, says Campbell, Jenkins used to plan train journeys, including times and interchanges, to faraway destinations, and could reel off all the stations on the Paris metro.

Harry Truman, too, was a train lover. Truman excelled in math as a boy. His entire life, he was often lonely, and the kind of man who “insisted that things be done just so,” concerning himself “with details of a kind others would never have bothered with” (see McCullough, 1992, quotations from p. 182). His communication skills were said to be so bad as to be “instructive mainly as a source of warnings” (Greenstein, 2009, p. 39). But when it came to trains, Truman was “fascinated.” As a boy, he used to sit alone on the roof and count the number of freight cars in each train rolling by. When asked, years later, if he remembered how much he used to love trains, he said, “I still do” (McCullough, 1992, p. 44).

A look at obsessive-compulsive (or “anankastic”) personality disorder shows that, like autistic persons, people of this type often want others to perform a task in exactly the right way. They also tend to be inflexible and obsessed with details (see Millon et al., 2004). However, they tend not to have the problems communicating and interacting socially that typify autistics (see, e.g., Baron-Cohen, 2008), and, it seems, many of the powerful people described here.

Finally, it’s worth mentioning that schizotypal personality disorder can also overlap with autism (e.g., Barneveld et al., 2011). The tendency to be cold, aloof, and withdrawn is a commonality. However, schizotypal types also often have abnormal perceptual experiences, magical thinking, and paranoia, among other things (American Psychiatric Association, 2013), signs and symptoms not typically found in autistics or powerful persons.

## Affable and Socially Talented?

It has long been a popular assumption that successful leaders are affable and socially talented (e.g., Caldwell and Wellman, 1926; Judge and Bono, 2000). Leadership scholars Goleman and Boyatzis (2008, p. 74) depict “great leaders,” and “place them on the opposite end of the neural continuum from people with serious social disorders, such as autism.”

Yet several powerful individuals have been deemed autistic by other authors, among them legendary leaders such as U.S. president Thomas Jefferson (Ledgin, 2000), French president Charles de Gaulle (Fitzgerald and O’Brien, 2007), and Irish president Éamon de Valera (Fitzgerald, 2004), as well as tyrants such as Vidkun Quisling, Norway’s WWII collaborationist prime minister (Dahl, 2012). Like other diagnoses from afar, these, too, must be viewed with skepticism – but they could lend some credence to a slightly less specific assumption, that powerful politicians such as these may have had more autistic traits than the average person. Let us look at one of them.

Charles de Gaulle thought a true leader should renounce friendship and isolate himself from his men, says his biographer (Fenby, 2010). “One does not exist in his eyes as a distinct person,” wrote Claude Mauriac, the president’s private secretary. De Gaulle, he continued, “judges what you say *in abstracto* without linking it to who one is or what one knows” (Mauriac, 1970, p. 167, translated by Jonathan Fenby). Control was everything to de Gaulle, says Fenby, who goes on to describe the president using words like “aloof” (p. 138), “cold” (p. 82), “distant” (p. 82), “compulsive” (p. 5), and “physically awkward” (p. 82), calling him a loner, ill at ease on social occasions, and with an explosive anger. De Gaulle’s command of the French language was extreme (Fenby, 2010), yet “foreign diplomats were often dumbfounded by his lack of verbal communication” (Haskew, 2011, p. 40). Anomalous communication and social interaction are typical of autistic people (Baron-Cohen, 2008).

Descriptions of other presidents can also cast light on the possible connection between power and autistic traits. South Africa’s Nelson Mandela, for instance, was often described as “aloof” (e.g., Roberts, 2013). Family members felt he was cold and unable to express love, describing instead the extent to which politics was an intense and circumscribed interest for Mandela: “He’s given his life to politics. He lives and breathes politics” (see Malone, 2013). Mandela’s daughter has been quoted as saying: “He’s awkward. To his children he’s awkward; he doesn’t know how to reach out” (see Malone, 2013). “[R]emote self-sufficiency,” were the words a biographer (Lodge, 2006, p. 17) used to describe the typical attitude of the liberation hero.

Mandela’s children, then, thought him a distant father – and “old friends teased him about his being persnickety and pedantic,” reported Roberts (2013). People with autism are often said to be pedantic (Attwood, 2015).

Mandela, whose record-keeping was “obsessive” (see Nkosi, 2015), was also obsessed with newspapers, said his wife, and after reading them he folded every paper exactly the same way every day. Each day he also took off his hearing aid, lined it up on the table, and then placed a badge he was wearing in position, and then his pens, so that “the pens, and everything has the same line” (see Roberts, 2008). Lining things up in rows or patterns is a common habit among people with autism (South et al., 2005). It appears Mandela was “structuring his life down to the smallest details” (Roberts, 2008). People with autism tend to be very attentive to details (Baron-Cohen et al., 2009).

Another African president, Robert Mugabe, was not popular with the children he grew up with (Robert Mugabe, 2007), who used to tease him (Holland, 2008). Teasing and bullying happens, alas, to most high-functioning children with autism (see Kaland, 2010). Mugabe was always a loner, says a biographer, an aloof man who wanted to make all decisions himself, and never made any friends (see Holland, 2008). He “lacked emotional understanding of social interaction,” (Holland, 2008, p. 74), sometimes refusing, for instance, to say a word in one-on-one meetings (Robert Mugabe, 2007). “Almost everybody who knows Robert Mugabe has commented on his coldness,” says Holland (2008, p. 182), who first met him in 1975, and noted later that he “he swaggered awkwardly as he does still” (see Heidi Holland, 2012). People with autism often have a strange gait (see Papadopoulos et al., 2014).

Let’s end this part with U.S. president Lyndon B. Johnson. He was a bossy child, as described by Caro (1982): If little Lyndon wasn’t allowed to be in charge, he would not take part in a game. As an adult, he still had a strong need to dominate. Autistic people are often bossy and controlling (see Baron-Cohen, 2008).

In discussions, Johnson tended to lecture – and always about politics (Caro, 1982). For Johnson, politics is described as being an intense and circumscribed interest from childhood. Even as a boy, Johnson was only happy when he was “politicking,” said his relatives (see Caro, 1982, p. 72).

Johnson was a perfectionist, a “very, very detailed” man for whom “things had to be absolutely correct,” said an old friend (see Caro, 1982, p. 354). This is also typical of many people with autism (Baron-Cohen, 2008). Caro (1982) goes on to describe the 36th president as intense and rigid, often worried and fearful, with periods of depression. Caro (1982) also often returns to his “lack of physical coordination” (p. 192), pointing out, among other things, Johnson’s “awkward walk” (p. 171).

## Did Power Change Them?

Lord Owen, a physician and former British foreign secretary, has described a condition he calls “hubris syndrome,” which he claims can befall the powerful. Reduced empathy and loss of contact with reality, often associated with progressive isolation, are among the signs and symptoms (Owen, 2015). Owen and Davidson (2009) assume that hubris syndrome will tend to abate once power is lost.

There is every reason to think that autistic traits exist from early childhood (e.g., Loveland, 2011). We have just seen, however, that feeling powerful can make a non-autistic subject seem more autistic-like. So when powerful people in real life seem somewhat autistic – have they always been that way, or did power change them? Not only Owen’s hubris syndrome, but also the priming studies discussed above might point to the latter alternative. A clear possibility, however, is that power does change people, but not completely. Indeed, achieving power might often strengthen autistic tendencies that were already there, already stronger than average. Our discussion of testosterone, below, will be relevant in this regard. Asperger (1944) claims that autistic traits can help people get high-status jobs. It is not a great leap to assume that some of those jobs will be positions of power.

## But Can Autistic Traits Be Helpful?

It may be, as our discussion indicates, that autistic traits do not necessarily prevent a person from achieving power. Moreover, it is interesting that so many autistic-like traits can be found in the powerful – their thinking styles, self-centeredness, preference for solitary activities, being socially distant, having less empathy, a reduced ability to recognize emotions, and several other things (see **Table 1**). However, the present paper’s hypothesis specifically says that autistic traits can be helpful in achieving power. We shall look at this in the sections to follow.

Thought-provoking data exist that can throw light on whether autistic traits can help propel a person (or another primate) to power. For instance, van de Waal et al. (2013) describe 10 male vervet monkeys who migrated into a new group. Nine of the 10 changed their diet, and started eating what the new group ate. One individual held out, however, and kept eating what he had always eaten. This inflexible, socially insensitive monkey quickly rose to take the top rank of the group he had migrated into.

Let’s continue, however, with a quick look at what humans need to acquire and sustain power. Does it take a gregarious and agreeable personality? Many studies now indicate that this is not the case (e.g., Collins and Hansen, 2011; Grant et al., 2011), and illustrative cases are legion. President Obama is generally said to be “aloof” (e.g., Bouie, 2013), dealing with foreign leaders and members of Congress in a “cool, businesslike” manner (Shear, 2015). “He’s not good with personal relationships; that’s not what interests him,” said a U.S. diplomat (see Cooper and Worth, 2012). Obama “has largely outsourced the relationship building,” says *The New York Times* (Shear, 2015).

Mitt Romney, Obama’s 2012 opponent, has been called “reserved by nature,” “data-driven,” and “emotionally remote,” with a “sometimes awkward style and aloof manner.” As governor of Massachusetts, “he wanted to control everything,” “tended not to socialize,” and tried to avoid casual conversations (see Barbaro, 2012).

Does acquiring and holding on to power take strong motivation? There are often more ambitious individuals than positions available, which would indicate that much motivation is needed. Where, then, could motivation to acquire power come from? We shall consider evolutionary mechanisms, but let us first look at proximate causes. Whether or not Ronald Reagan had more autistic traits than the average person, he was seen as having a need “to impose order on chaos” (Morris, 2004) – and this may be a good place to start.

Having autistic traits tends to mean feeling a need for control and orderliness (Kanner, 1943; Overskeid, 2016). That need may make you try to order the physical world, as in scientific research – and people with autistic traits seem especially drawn to the physical sciences (Baron-Cohen et al., 2001). Yet holding social power may well mean having even more control.

Angela Merkel, the German chancellor, could be a case in point. A biographical portrait (Radice, 2013) pointed out that “she always wanted to use power. It’s just that before the [Berlin] wall came down, she wanted power over molecules… and after the wall came down, it was power on a wider stage.” Merkel studied physics and holds a doctoral degree (Lachmann and Reuth, 2013). People in the “hard” sciences tend to have more autistic traits than others (Austin, 2005).

As a girl, Merkel took part in the “Math Olympics” (Müller-Lissner, 2015), and knew all the names of members of the East German as well as the West German governments, but still “… tried not to be a nerd” (Mills, 2008, p. 25). She was also physically clumsy, and has explained: “What a normal person can do quite naturally, I had to process consciously and perform laboriously. I was a little idiot when it came to moving. My patient parents always had to tell me how to walk down a hill – from a technical point of view” (see Leßnerkraus, 2005, p. 97, my translation). An associate who has known her long says Merkel is “very difficult to know … She is like a computer …” (see Packer, 2014). In political meetings, “she is almost uncannily immersed in the details of whatever is under discussion” (Irwin, 2015). Other observers have called Merkel “remote,” “a bit awkward,” and noted her “analytical detachment” (see Packer, 2014). High-functioning people with autism are often seen as analytical (Bryson, 2005), and can be socially detached (Soderstrom et al., 2002).

An important reason for the success of a surprising number of people with many autistic traits, said Asperger (1944), is the fact that people in this group tend to concentrate strongly on a special interest, which he thought could result in great accomplishments. And when those tending toward autism choose politics, perhaps we should not be so surprised. Politics has been called “a hypermasculine world” (Brooks, 2015, p. 46), and autism is, after all, seen by some as hypermasculinity (Asperger, 1944; Baron-Cohen, 2003).

It was perhaps to be expected, then, when researchers found autistic people to often be controlling and bossy (Baron-Cohen, 2008), with an intense and narrow focus on the goals or interests that preoccupy them (see Bryson, 2005; Smith et al., 2009), and ofttimes extremely persistent (Gillberg, 2002). This combination of traits may help people get ahead in many areas. Regarding *where* these individuals might want to get ahead, it is interesting to note that many individuals with autism do appear to take a strong interest in politics (see, e.g., Baron-Cohen et al., 1999; Walker and Fitzgerald, 2006; Palermo, 2013; Government Equalities Office, 2014). So when high-functioning autistic people are asked about their interests, science and gadgets are not the only subjects they mention – but also politics and closely related areas, such as the U.S. Congress, U.S. presidents, history, and social sciences (South et al., 2005; Klin et al., 2007; Lorenz and Heinitz, 2014). It does not seem unthinkable, then, that people with many autistic traits will try their luck in politics, and that some will reach positions of power.

## Buy A Dog

Autistic traits that contribute to persistence and a strong focus on goals can increase one’s chances of reaching ambitious objectives. To most people, however, another consideration is also important: we want to be liked. At a minimum we want to avoid being disliked. On the other hand, achieving much social power is well-nigh impossible if one is too sensitive to others’ real or imagined negative reactions. There is competition for positions, and it is difficult to be popular among those one tries to beat and their allies. Also, having won a position of power, one must make decisions that will often be opposed.

“If you want a friend in Washington, buy a dog,” is a tip that comes not only from kennel owners, but also from politicians (see, e.g., Fisher, 1987). Donald Rumsfeld has added: “Better make it a small dog, because it may turn on you also” (see Safire, 2008, p. 635). People observing politics outside of Washington have reached similar conclusions, for instance Machiavelli (1966), in renaissance Italy, and Manmohan Singh, the former prime minister, in present day India (see PM hints at, 2013).

Not unexpectedly, then, many powerful people have been rather lonely figures (Ludwig, 2002). Among those described with words to that effect are presidents like Washington [“had no friends in the real sense of the word” (Ferling, 1988/2010, p. 262)], Polk [“… not well liked … He had few close friends (Byrnes, 2001, p. 157)], Truman [“never popular like other boys” (McCullough, 1992, p. 35); often felt “lonely” (McCullough, 1992, e.g., p. 1042)], and Carter [“as a boy, and later as a man, Jimmy was ‘lonely”’ (Godbold, 2010, p. 22)]. Legendary Polish president Józef Piłsudski was “a loner” (Hetherington, 2012, p. 357), as was West German chancellor Willy Brandt [“an utter lack of intimates … a loner” (Drath, 2005, p. 15)], and also recent British prime ministers Thatcher [“She had no real friends” (Campbell, 2009, p. 367), Wilson (“general lack of need or gift for friendship” (Jenkins, 2004)], and Heath [“introverted and lonely man” (Ziegler, 2011, p. 512), Israeli prime minister David Ben-Gurion (“made few friends” (Shapira, 2014, p. 54)], Danish prime minister Jens Otto Krag [“did not make many friends – if any” (Pedersen, 1995, p. 255)], and Scottish first minister Alex Salmond [“always… something of a lone operator” (Wheeler, 2014)]. This list must have an end, but finding more examples is not difficult (e.g., Ludwig, 2002). Those with many autistic traits also tend not to have many friends (Lasgaard et al., 2010; Ingersoll and Wainer, 2014).

It should perhaps not surprise us, then, that powerful people often act in ways that do not normally further friendly relations. As we saw above: They tend to be more self-centered than others (Galinsky et al., 2006), more socially distant (Lammers et al., 2012), more often treat others as objects (Lammers and Stapel, 2011), and have a stronger preference for solitary activities (Conlon, 2012). Many such characteristics we saw the powerful share with those who have autism (see **Table 1**).

A lack of interest in others and what they might think could leave you rather lonely, but such lack of interest need not be a handicap for the person wanting to gain and hold on to power – and it could be an advantage. Galinsky et al. (2008, p. 1463) point out that “the experience of power liberates individuals from the straightjacket of the social world, allowing them to define for themselves what is and is not achievable…. [I]t is the protection from situational influence … that helps powerful individuals surmount social obstacles and reach greater heights of creativity to express the unpopular ideals of today that can lead others to the horizons of tomorrow.”

And there is more. In explaining Gordon Brown’s success as a negotiator, a senior official said: “Gordon is stronger because he doesn’t care whether people hate him …” (see Rawnsley, 2010, p. 58). Mount (2011) described another British prime minister, Harold Macmillan: “[T]hroughout his life he was disliked by many and hated by quite a few…. Not that he much minded being unpopular.” Autistic people often seem unconcerned regarding what others might think of them (Izuma et al., 2011).

It is also worth contemplating that even in animal leaders, lack of social sensitivity is widespread (e.g., van de Waal et al., 2013). Johnstone and Manica (2011) see leaders across types of animals as stubborn, mostly acting the way they are themselves inclined to, not responding much to other group members. Interestingly, in humans, the most effective leaders appear to be those who do *not* behave in accordance with the cultural norms of the organizations they lead (Hartnell et al., 2016). Autistic children, says Asperger (1961, p. 182), “in all things follow their own impulses and interests. Unconcerned by others’ wishes, they say and do exactly what they want” (my translation).

Across types of animals, there are many indications, then, that those who acquire power are often the most stubborn individuals – those who paddle their own canoes, not those most sensitive to others’ needs or feelings (Conradt et al., 2009; Johnstone and Manica, 2011; van Vugt, 2014). This could of course be different in humans – but it does not seem to be. We saw Gardner (1995) pointing out that those who go on to be leaders often confront authority from an early age. In Britain, the most rebellious member of Parliament for the 13 years that Labor was last in government, voting against his own party 428 times, was Jeremy Corbyn (Cowley, 2015). He is now the leader of the Labor party. Other recent British party leaders, Labor as well as Conservative, have also been rebellious in this sense – examples being Neil Kinnock and Iain Duncan Smith (Cowley, 2015).

Features powerful humans seem to share with autistics also have to do with insensitivity: we saw the diminished capacity to read others’ emotions, the reduction in empathy or empathic accuracy, the bossy behavior. Autistic children, says Asperger (1961, p. 188), feel a “need to always go their own way and use methods they have themselves invented” (my translation).

Powerful persons make decisions affecting many lives, and must sometimes even place others in harm’s way. In such circumstances, empathy can be a poor guide to ethically sound decision making (Bloom, 2013). Instead, the best decisions will often be made by following utilitarian ethical rules, instead of trying to empathize with all affected individuals. It is, for example, a common view that Congress was ethically right in declaring war on Germany in 1941. Yet too much empathy with those bound to suffer and die in that war might have made the decision difficult, though it ultimately helped rid the world of Nazism and thus prevented wretchedness affecting even more individuals. We saw that autistics and the powerful tend more often to make rule-based moral choices than do other people (Lammers and Stapel, 2009; Baron-Cohen, 2011). Interestingly, Lucas and Sheeran (2006) assume that Jeremy Bentham, the founder of utilitarianism, had Asperger syndrome.

In less dramatic situations, too, a powerful person may have few alternatives other than treating others as tools or as objects. In filling a post, you want the most qualified individual, even if another applicant would be bitterly disappointed by not getting the job. In other words, you want the best human tool, not the person who might most need your empathy. It is probably also a fact that those aiming for powerful positions may find that it necessary to use people and hurt feelings in order to satisfy their ambition. This, too, will likely be easier for those with some degree of social insensitivity. Hauser (2013, p. 20) goes further, claiming that a “tendency toward cruelty has been selected because it can help achieve and sustain power by striking fear in others.”

Many powerful people have been described in ways that are consistent with a lack of social sensitivity. If we limit ourselves to some early American presidents and the most recent British prime ministers, we see that John Quincy Adams was supposedly “famous for his cold demeanor” (DeGregorio, 2009, p. 89), that James K. Polk appeared “cold” (Byrnes, 2001, p. 157), and that Lincoln was seen as a “cold man” (see Burlingame, 2008, p. 181) – while David Cameron “can be ruthless” (Theakston, 2015, p. 7), and Gordon Brown, as we saw, was said to operate with “Stalinist ruthlessness” (Timmins, 2007). Autistic children, says Asperger (1961, p. 191) are “ruthlessly intent on enforcing their wishes” (my translation).

Now achieving power and keeping it are two different things. It seems reasonable, given the relevant data, that holding power can increase the tendency to make cold decisions (Lammers and Stapel, 2011), and be more self-centered (Galinsky et al., 2006) and less sensitive (among other things, see Hirsh et al., 2011). But though that may be true, we should perhaps assume that on their way up, those who aren’t yet very powerful would have much to gain by being warm, other-centered, and sensitive to the feelings of people around them.

Or maybe not. By the time Lyndon B. Johnson was a college student, he showed signs of limited empathy. He used threats to win a student election, and to have fun, he tricked an acquaintance into putting cow dung on his face, then telling others so they could ridicule the boy (Caro, 1982). At the same time Johnson flattered the college president so intensely that the president “displayed toward Lyndon a friendliness he had never displayed to any other student …” (Caro, 1982, p. 192).

Much genuine empathy may not be necessary to rise in an organization. And we saw that even psychopaths can have impressive careers, at least in the corporate world (e.g., Babiak et al., 2010). There is also evidence that following a simple rule of flattering those you want to influence can get you a long way (Westphal and Stern, 2007) – and there is no doubt that people with many autistic traits can learn and practice complex sets of rules to master social interaction. It seems clear that those who have Asperger syndrome can do it (Baron-Cohen, 2003), and they get better as they get older (Asperger, 1961). Schwenck et al. (2012) found continuous improvement in the empathic skills of autistic people through childhood and adolescence.

Furthermore, Baron-Cohen et al. (1999, p. 477) point out that “a good understanding of politics” may be “independent of the kind of empathic skills involved in more intimate social relationships.” And even with the social handicaps that come with autism, Asperger (1944, p. 117, my translation) noted that “these children … often make astonishingly precise and mature judgments of the people around them.” And since our focus, after all, is primarily on people who have many autistic traits without quite deserving a diagnosis, it is interesting to see that the jury is still out with regard to which (if any) deficiencies this group has when it comes to understanding other minds. For example, Baron-Cohen and Hammer (1997) found that parents of children with Asperger syndrome did worse than controls on a theory of mind-related test – while in a study of healthy university students, Kunihira et al. (2006) did not find a relation between results on a theory of mind test and a test of autistic tendencies – and Henderson et al. (2015) found no difference between high functioning autistic participants and typical controls on theory of mind tasks.

Finally, the extent to which autistic people’s typically low scores on tests of theory of mind are caused by low motivation rather than low ability is also unclear. It does appear, however, that when the setting is competitive, many children with autism can track the beliefs of other individuals, thus indicating that, at least in this context, they have a theory of mind – even if the same kids fail traditional tests designed to gauge this construct (Peterson et al., 2013, see also Chang and Cheung, 2016). Politics, of course, is often very competitive, and might therefore also motivate theorizing about the minds of others.

As is normally the case in autism, we also saw that taking others’ perspective seems difficult for people who feel powerful (Galinsky et al., 2006). But this, too, appears to change when participants need to take others’ perspective to succeed in a task they have set out to accomplish. Then subjects primed with high power can do even better than those primed with low power (Schmid Mast et al., 2009).

## Competence

Whether voters voice considered opinions or form quick, automatic judgments, they appear to want competent political leaders (Bull, 2005; Todorov et al., 2005). So let us look at relevant competence in high-functioning autistics. One aspect is memory: the autistic person will often have an impressive ability to remember facts, especially in his or her areas of special interest (Kanner, 1943; Asperger, 1944) – and to become powerful in competitive fields like business or politics, business or politics must probably be your special interest. Moreover, not only do autistic people often have impressive memories, they also seem better than neurotypicals at distinguishing false from real memories (e.g., Beversdorf et al., 2000; Hillier et al., 2007).

But memory is not enough, other aspects of intelligence are also needed, and high-functioning people with autism appear to score higher on fluid intelligence than do controls matched for gender, age, and full IQ (Hayashi et al., 2008).

Specific thinking skills are certainly also important. There is evidence that those who have autism are able to think in more logical and less biased ways than typical controls with similar IQs (De Martino et al., 2008); this also includes being better at drawing conclusions regarding causes and mechanisms (Baron-Cohen et al., 1986). Autistics are apparently also less prone to jumping to conclusions (Brosnan et al., 2014), and to committing a common fallacy (Morsanyi et al., 2010). In addition, autistics and people with subthreshold autistic traits seem better than others at thinking creatively (Kasirer and Mashal, 2014; Best et al., 2015).

Intelligence, creativity, and thinking skills can help people reach many goals, and it would be surprising if such attributes were irrelevant to achieving power. Given the competence often found in those who have many autistic traits, it may not be surprising that there could be more such persons among the powerful than we had perhaps expected.

## But Aren’t Autistic People Often Anxious, Depressed, and Powerless?

People who have many autistic traits, sometimes referred to as the BAP, are assumed to have many characteristics in common with those who have autism (e.g., Ingersoll et al., 2011). And we should not forget that much more often than other people, autistic people have various psychiatric problems, such as anxiety – which also appears to be the case in those who simply have many autistic traits (Kunihira et al., 2006).

Anxiety, especially if frequent and severe, can make it difficult to function well in any job. Difficulties can be overcome, however, and a number of people have performed well in powerful positions while battling angst and apprehension – three examples being British prime ministers Stanley Baldwin (Thorpe, 1998), Anthony Eden (Davidson, 2011) and Harold Macmillan (Mount, 2011). Hence it does not seem that being anxious rules out gaining and sustaining power – nor, apparently, does having depression, which may be more common among the powerful than anxiety (e.g., Davidson, 2011). There is some evidence, even, that tending toward depression need not be a disadvantage to one who wants power, and could even be an asset, especially if paired with periods of elevated mood (e.g., Kyaga et al., 2015). Let us look a little closer, then, at the relations among autism, power, and fear, which have some intriguing aspects.

First, while there is no doubt that autism often implies anxiety and depression, autistics may also seem fearless (Bachevalier and Loveland, 2006) – as may powerful people. Indeed, Winston Churchill, while suffering from recurrent depressions (see Davidson, 2011), proved time and again his lack of fear, which more than once gave rise to foolhardiness (Arnett, 1991). De Gaulle “suffered from periodic moods of depression” (Fenby, 2010, p. 415), and said his life had been “fairly filled with worries and melancholy” (see Fenby, 2010, p. 349), yet “[h]is habitual fearlessness was evident to all …” (Fenby, 2010, p. 125).

We saw above that Harold Macmillan had nervous breakdowns (Mount, 2011) and recurrent depressions (Davidson, 2011). Yet as a soldier in World War I, he showed conspicuous bravery (Matthew, 2011). Later, himself wounded in a plane crash, Macmillan risked his life to save another man (Thorpe, 2010).

The examples of Churchill, de Gaulle, and Macmillan should be sufficient to show that powerful people can be in far from perfect psychological health, but still tolerate substantial risks when needed, which is presumably often necessary for those who seek and hold power.

The extent to which high risk tolerance is important for acquiring and holding on to power is important, and not yet resolved. For example, the exceptionally successful leaders studied by Collins and Hansen (2011) were called “neurotic” (p. 30), and said to show “hypervigilance” at all times (p. 29). According to Collins and Hansen (2011, p. 27), these leaders never felt safe, and were “afraid – terrified, even – of what the world [might] throw at them.”

It seems, then, that people with some anxiety, perhaps even a good deal, can acquire power and keep it for some time. There is even the possibility that tending toward anxiety can help a person’s career – it may be smart, for instance, to never fully relax in an ultra-competitive environment. On the other hand, there is much evidence indicating that neuroticism in general and anxiety in particular may be unhelpful to someone seeking power (e.g., Stein and Kean, 2000; Moutafi et al., 2007).

Most of what we think we know about autism comes from people who need help. They come into contact with psychologists and doctors and hence become their research subjects. These people often have other problems in addition to autism, such as anxiety or attention deficit disorder (Montes and Halterman, 2006; White et al., 2009). It might be that undiagnosed autistics have less depression and anxiety.

Then again, we saw above that the group we are most interested in (the BAP, that is, those with subclinical autistic traits), have many of the characteristics often found in autistic people – anxiety prominent among them (Kunihira et al., 2006; Wainer et al., 2011).

An interim conclusion, then, would be that some anxiety could at times be helpful to those wanting power. Yet anxiety and other problems that often bother autistics can very probably also hinder the power-hungry.

In the average person, lack of power can lead to anxiety (see Fiske et al., 1996). There is much evidence indicating that being in control feels especially important to autistics (see Kanner, 1943; Lyons and Fitzgerald, 2005). And since the connection between power and social control is so intimate – could it be that lack of power feels especially anxiety-provoking to those who have many autistic traits? And might it also be that such anxiety could be one source of motivation for power-seeking in people with many autistic traits? Josephs et al. (2006) found that people high in baseline testosterone (T) reported increased emotional distress when placed in a low status position, while those low in baseline T thought being placed in a high status position was distressing – and there is much evidence that testosterone and other androgenic hormones plays a role in occasioning autistic and power-related behavior.

## Testosterone As A Common Cause

It may well be important that autistic and powerful people seem to share many traits. Yet this remains a correlation. A stronger test of the claim that powerful people have more traits that can really be called autistic, would be to look for a common mechanism causing the shared traits. Interestingly, one appears to exist.

All humans produce testosterone (T), but in different amounts depending on sex, individual differences, and possible diseases (see Auyeung et al., 2013). Significant quantities of T originate in the testes, with the ovaries and adrenal glands as other sources (see Puri, 2011), while a preponderance of the brain’s relevant receptors can be found in a subset of hypothalamic neurons (Fernández-Guasti et al., 2000).

T helps shape thinking, feeling, and behavior in specific ways. This takes place in concert with other hormones and neurotransmitters, but T’s role is important. Indeed, van Honk et al. (2014, p. 201) argue that “the social brain’s main chemical, without exaggeration, is testosterone.” One’s current stage of development helps determine testosterone levels (see Auyeung et al., 2013), as do life events, such as social success or defeat (see Eisenegger et al., 2011).

T starts shaping the brain in the womb, a point in time at which the amount of the hormone available is important in deciding the relatively permanent organization of several aspects of the brain. The amount of T available prenatally can vary greatly as a function of sex, and also among individuals of the same sex (Lombardo et al., 2012). After birth, varying levels of circulating testosterone can also activate tendencies to think, act, and feel in specific ways (see van Honk et al., 2014).

### Testosterone and Behavior

Since autism is much more prevalent among males, but not restricted to that sex, it is not surprising that several researchers have focused on androgenic hormones, and testosterone in particular, as possible causative factors. Some findings indicate that adult persons with autism, perhaps especially women, have excessive androgen levels (e.g., Geier and Geier, 2007; Ruta et al., 2011; Schwarz et al., 2011; El-Baz et al., 2014). Yet there are more studies to back the assumption that high prenatal (i.e., intrauterine) androgen levels increase the probability of autism (e.g., Baron-Cohen et al., 2015). The one, of course, does not rule out the other. According to James (2014, p. 37): “The evidence is overwhelming for Baron-Cohen’s hypothesis that one cause of autism is exposure to high levels of intrauterine testosterone.” Many studies indicate that administering T to healthy adults of both sexes will increase the probability of several behaviors associated with autism as well as with the feeling of being powerful (see, e.g., **Table 1**).

And what kind of person do we talk about when we talk about someone affected more strongly than most by T? Among other things, this individual would tend to be detail-oriented (Auyeung et al., 2009), bossy or dominant (Turan et al., 2014), self-centered (Mehta et al., 2009; Wright et al., 2012), and often lonely and socially distant (Turan et al., 2014). The person would have difficulty reading others’ emotions (Ronay and Carney, 2013), and be low on empathy and empathic accuracy (see Auyeung et al., 2013; Ronay and Carney, 2013). He (or she) would probably be somewhat insensitive to social consequences (van Honk and Schutter, 2007), but often quite persistent (Welker and Carré, 2015), and, at least if a boy, with a strong and narrow focus on goals (Auyeung et al., 2013). **Table 1** shows that most of these behaviors (and others) can be found in powerful people, as well as in those with many autistic traits.

If authors such as Auyeung and Baron-Cohen (2013) and James (2014) are right, then, high prenatal T levels are an important cause behind autistic traits. And if their brain sare shaped by T – assumed to be the hormone of social dominance (van Honk et al., 2014) – it is not surprising that those with many autistic traits behave in ways that can help them achieve positions of power (see **Table 1**).

Since behavior like that of the powerful and those with many autistic traits has also been shown to result from T, it is interesting to note that the effect of T administration is especially strong in people exposed to high prenatal levels of T (van Honk et al., 2011; Carré et al., 2015).

It seems a reasonable assumption, then, that if a person exposed to high levels of prenatal T achieves power as an adult, traits that helped him or her achieve that power (many of which may well be autistic traits) will be strengthened, perhaps considerably, by the increased level of circulating T that tends to result from feeling powerful. Several studies have now shown that making people feel dominant, powerful, or victorious will tend to increase T levels (e.g., Bernhardt et al., 1998).

## Coincidental Commonalities?

The many characteristics common to autistics and the powerful may not be a coincidence, then. There are indications, as we saw, that autistic behavior is caused, in part, by T’s effect on the brain (Schwarz et al., 2011; James, 2014). Furthermore, there is evidence that feeling powerful can increase T levels (see Mehta and Josephs, 2011), and that T can increase the tendency to act in ways that are typical of powerful people (e.g., van Honk et al., 2011).

In a literature review, Mehta and Josephs (2011, p. 176) concluded that people “high in basal T are chronically motivated to gain status.” There are many similarities between those who presently have a high basal T level and autistics, whose prenatal brain organization has presumably been affected by T to a greater extent than that of most people (see **Table 1**). For instance, Sapienza et al. (2009) found that the effect of T measured in saliva (hence being present in the body here and now) was quite similar to the effect of T measured by way of markers of prenatal exposure.

So if people high in basal T are chronically motivated to gain status, it is not unthinkable that autistic people are also motivated to gain status, perhaps more so than others. To the extent that status is a means to achieving power or control, that assumption is supported by the finding that people with many autistic traits have a strong need to be in control (e.g., Lyons and Fitzgerald, 2005).

## Dominant or Alienated?

Here’s a good question: If T gives rise to dominant behavior, why are autistic people often, in the words of Baron-Cohen (2008, p. 10), “struggling with… feelings of marginalization, alienated on a planet where they do not feel they belong?” Part of the answer begins with Baron-Cohen (2008, p. 79) pointing out in the same book, that “bossy, controlling behavior” is also typical of autistic people. This reflects what we have already seen: It is possible to often feel wretched and still behave in a domineering manner.

Furthermore, we are still primarily discussing people who do not qualify for a diagnosis. Presumably, those who have subclinical autistic traits do not struggle with problems that are quite as big as those of real autistics. Moreover, the extent to which effects of high T levels in adults parallel those arising from high prenatal T levels still is not clear. It is interesting, however, that T appears to give rise to dominant behavior only in people whose cortisol level is low. Combined with a high cortisol level, T does not seem to occasion dominance. Indeed, it may make people behave in less dominant ways (e.g., Mehta and Josephs, 2010). Those who have many autistic traits are often plagued by feelings of stress and anxiety (White et al., 2009), feelings known to be correlated with heightened levels of cortisol (Hamer et al., 2012). This is one potential reason why people with many autistic traits may not be dominating at all.

## The Need to Take Risks

Several disorders are thought to exist because there has been, and perhaps still is, selection for genes that promote certain characteristics. Nesse and Williams (1998) give the sickle cell gene as an example. One copy of the gene will protect against malaria, and is thus selected for where that disease is prevalent – even though those who inherit two copies will get sickle cell anemia, a painful and often deadly disease.

No single gene lies behind autism (Chahrour et al., 2012). It still makes sense, as pointed out by Reser (2011), to assume that autism has persisted because genes that help cause it have provided some advantage. The hypothesis advanced in the present paper is based on this assumption, which has been voiced by several authors (e.g., Del Giudice et al., 2010; Reser, 2011). The nature of the hypothetical advantage conferred by autistic genes is unclear, however.

Researchers such as Baron-Cohen (2003) and Baumeister (2010) have pointed out the possible advantages of typically male characteristics in acquiring power. Indeed, in discussing the brain of typical human males, Baron-Cohen (2003) notes, among other things, how low empathizing and good systemizing abilities could lead to power, which could in turn bring reproductive success – and in doing so, Baron-Cohen (2003) gives several examples similar to those in the present paper. As regards autistic traits and fitness, however, Baron-Cohen and his co-workers assume that autistic traits may have been selected for “because of the potential benefits of a solitary single-minded obsessive focus on innovative understanding of a system,” which could help a person acquire resources by way of his or her “building and fixing skills,” or via trade in products (Lai et al., 2014, p. 903).

It is possible, however, that hypermasculinity resembling autism can sometimes be an advantage in the struggle for power. Competition is stiff; opponents intelligent, driven, and often insensitive. The behavior pattern known as the BAP is, it appears, an adaptation that increases the probability of strong motivation, a willingness to run risks, and test smart, unconventional thinking – which for some individuals will result in power and more procreation.

In describing the phenomenon of sexual selection, Darwin (1871) pointed out that there is competition within each sex for reproductive access to the most desirable members of the other sex. He also noted that in most species, males tend to be “more eager” to engage in copulation (Darwin, 1871, p. 272). This, it seems, includes the human male (Baumeister et al., 2001).

Bateman (1948) called attention to the fact that females tend to choose among competing males, and that fewer males than females thus get a chance to procreate, leading to stronger competition among males. The range and variance in reproductive success has thus been considerably larger for men than for women (Betzig, 2012).

Baumeister (2010) points out some relevant facts: humans alive today are descended from twice as many women as men. Estimates may vary, but throughout history, around 80% of women but only 40% of men have reproduced. Getting access to females in the face of intra-sex competition is not always easy for a man, in other words. But the problem can be solved by acquiring power.

There are several ways in which power can reduce or eliminate competition from other men. President Mobutu of Zaire, for example, was offered virgins from local chiefs on his travels around the country (Van Reybrouck, 2014). Men are also known to have used what power they have had to rape or buy women.

The harem is another alternative. Powerful men have kept groups of concubines in many different cultures, such as Turkey (Goodwin, 1997), Arabia (Olin, 1843), India and China (Betzig, 2012), as well as Africa, pre-Columbian America, and other places (Betzig, 1986). European kings have tended not to have harems, but they have had many illegitimate children, and also often powerful descendants through their misbegotten progeny – among them David Cameron, the recently departed British prime minister [descended from William IV (Heritage, 2014)], and, indeed, his wife [descended from Charles II (McSmith, 2011)].

A third way in which power can reduce competition from other men is by making sure one’s power is well known – and, given the typical female preference for men with power and power-related qualities (Buss, 1989; Fieder and Huber, 2012), chances are that, other things equal, potential mates will rather bear the child of a powerful man than that of lesser rivals.

There is evidence that men take more risks than do women, a difference affected by testosterone (Sapienza et al., 2009). In the right circumstances, most men are willing to risk life and limb – if their country is at war, for example. But “[f]ew men,” noted Kennedy (1966) “are willing to brave the disapproval of their fellows, the censure of their colleagues, the wrath of their society.” Those who have many autistic traits more often appear to take this latter risk, however.

Our discussion so far leads to the conjecture that nature creates people willing to brave the disapproval of their fellows by reducing their sensitivity to socially mediated punishment – resulting in traits that make you “trust yourself when all men doubt you” in the words of Kipling (1910, p. 181). In the poem, “If –,” from which the line is taken, Kipling describes an ideal man – a figure that evokes the thought of hypermasculinity, described by Asperger (1944) and Baron-Cohen (2003) as the essence of autism. Indeed, to trust yourself when all men doubt you is what you do when your primary reference point is yourself, which is often the case in autistics (Baron-Cohen, 2005), in whom context, social or otherwise, tends not to matter much (Vermeulen, 2012).

An independent outlook, sometimes fiercely so, is needed for creative thinking, for “defying the crowd,” to use the title of Sternberg and Lubart’s (1995) fascinating treatise. From their research on mental effects of power, Galinsky et al. (2008, p. 1463) conclude: “For the powerful, the situation recedes, and they are left with their own opinions, beliefs, attitudes, and personalities to drive their behavior. This receding of the situation may be a springboard for new ways of thinking.”

New ways of thinking can make the powerful take great risk, then, risk that can result in loss of social position, and sometimes of life itself. Fridtjof Nansen, the Norwegian scientist, explorer, and diplomat, is one such example. Starting out in biology, Nansen (1887), took great intellectual risk, “presaged Ramon y Cajal’s famous formulation of the Neuron Doctrine” (Edwards, 2006, p. 288), and “altered the course of neuroscience” (Compston, 2010, p. 2173). Nansen then found fame through perilous polar expeditions, before taking on a political role, first “leading [his] people to self-respect and independence” (Thyvold, 2011, p. 3), then becoming an internationally leading diplomat and the first high commissioner for refugees, taking more risk in saving thousands of lives – not just from behind a desk, but also on the ground, typhus claiming some of his closest collaborators (Stang, 1922). In 1930, the front page of *The New York Times* said “NANSEN DIES AT 68…” (Nansen dies at 68 of heart paralysis, 1930) “His outstanding figure shines in history,” said *Nature*’s obituary (Mill, 1930, p. 933). Called “father of the nation” (Skouen, 1991, p. 4), “Norwegians voted Nansen the greatest figure of the 20th century. So could we all,” wrote Britain’s *The Guardian* newspaper (In praise of …, 2008).

Nansen said he never had friends (see Karlsen, 2013), and was called “too self-centerd to be anyone’s friend … unsociable and clumsy” (see Huntford, 2001, p. 335). Not a team player, “in everything he … did, he led,” said Kobak (1999). Nansen’s brother remembered him simply as “the boss” (Huntford, 2001, p. 11). A “driven and tormented man,” said Huntford (2001, p. 668) his biographer, who also called him a “tortured, isolated polymath” (p. 586) with a “flawed insight into natures other than his own” (p. 635). “[S]eriously obsessed by my work …,” was Nansen’s self-description (quoted in Karlsen, 2013, my translation).

Maybe somewhat inflexible, “my trouble in life,” Nansen wrote, “is that I have never been able to compromise” (quoted in Huntford, 2001, p. 455). Seeming socially insensitive, he also wrote: “not worrying about the impression I make on others, I often insult those I least want to insult” (quoted in Karlsen, 2013, my translation). And politically, “to a man like Nansen, it meant nothing that the positions he took made him unpopular” (Kleivan, 1961, p. 451, my translation). Nansen had, he explained himself, “the habit of making up my mind without asking the opinion of others” (Nansen, 1926).

We have seen similar traits in other powerful persons, not least the apparent lack of interest in what others might think or feel – and we find the same insensitivity in many creative scholars. Isaac Newton, Albert Einstein, and Bertrand Russell are just three examples. The tendency is evident in their fresh and original thinking, but also in an often unfeeling manner, as their biographies show (Monk, 2000; Isaacson, 2007; Keynes, 2008).

Nature could (and does) create many people that are flexible, take others’ judgments into account, and adapt to societal norms. Among other things, such norms tend to regulate how to act, and what we should hold to be morally and factually right. Follow norms, and chances are you’ll live a reasonably safe and unremarkable life. If you are a woman, there is also a good chance that you will find a mate. For men who have thus chosen to play it safe, however, the chance of procreating has historically been considerably lower than in women choosing the same strategy (see Baumeister, 2010).

Take risks, break with norms, and you could find yourself in prison (or worse) – but with the right upbringing, luck, and intelligence, you could also be Charles de Gaulle, Bertrand Russell, or Nelson Mandela (though they, too, all spent time in prison)^2^.

Focus narrowly and perhaps obsessively on your goal, be inflexible and blind to others’ judgments, and you could win big or lose big. If losing means not procreating, chances are you would not have done so anyway. If you win, your descendants could be many. van Vugt (2014) points out that those who lead chiefdoms, kingdoms, and empires have greater reproductive success than does their average subject, and that male leaders in Western organizations have more sexual encounters than do men of lower rank.

Elon Musk, today a powerful businessman and the founder of PayPal, SpaceX, and Tesla Motors, was bullied so violently as a boy that at one point, he spent 2 weeks in hospital (Hogg, 2015). Musk tends to “come off as aloof and hard,” says his biographer (Vance, 2015, p. 363), and his communication skills may not be so good (e.g., Koetsier, 2015). He is also described as being socially insensitive, with intense and restricted interests (Vance, 2015).

If you are insensitive to social signals, you can easily become a bit peculiar, and peculiar people are often bullied, as are those who have many autistic traits (Baron-Cohen, 2008). But that same insensitivity can help you think creative thoughts, as we have seen. Creative thinking and intense interests can give you power, and Musk not only has power, but also six sons (Musk, 2010).

“Remember,” says Baumeister (2007), “most of the mediocre men left no descendants at all.” Indeed, in societies spanning from hunter-gatherers to the most modern, there is a positive association between reproduction in men and power-related characteristics like wealth and status. An important reason appears to be that men with low income are often childless (see Huber et al., 2010).

At the same time, there is little doubt that powerful men have had many children. Sultan Moulay Ismael of Morocco, for example, may well have had around 1170 children with more than 500 women (Oberzaucher and Grammer, 2014). In a large region of Asia, genetic analysis indicates that about 8% of all men today are descendants of Genghis Khan (Zerjal et al., 2003). Consistent with this, a recent study showed that “male descendants of high-status males account for a disproportionately large share of the male population in later generations” (Song et al., 2015, p. 574). It thus appears that people alive today are disproportionately the descendants of powerful men.

## Women

Indications seem strong, then, that a tendency toward autistic-like behavior has been selected because it has increased the probability of achieving power, and hence reproductive success. Testosterone appears to have been an important tool evolution has used to generate the behavior known as autistic – and power related – traits.

But what about women? Though the sex ratio is skewed, there is no doubt that many women have many autistic traits. I have argued that a subgroup of men with many autistic traits have hit the evolutionary jackpot by becoming powerful and have thus had many descendants. Is there a subgroup among women with many autistic traits who have also gained power and have had more children as a consequence?

Though the issue is controversial, it is possible that women who gain power are more like men or become more like them (e.g., Oshagbemi and Gill, 2003; Andersen and Hansson, 2011), which would mean having more autistic traits than other women (cf. Baron-Cohen, 2003). Moreover, the literature on power priming indicates that the effect is very similar in the two sexes (e.g., Anderson and Berdahl, 2002; Inesi et al., 2012), as is the effect of T, in the sense that the hormone appears to masculinize individuals of both sexes (Morris et al., 2004). It is definitely possible, then – even probable – that autistic traits can increase women’s chance of gaining power, as it can men’s.

Margaret Thatcher (see, e.g., Major, 2000; British Broadcasting Corporation, 2011) and Angela Merkel (see above), are two very powerful women who appear to resemble many equally powerful men when it comes to autistic-like traits. At least, this does not speak against the hypothesis that powerful women and men can be similar in important respects. An important question remains, however: Is there any reason to assume that being powerful has increased women’s reproductive success? I cannot see that there is.

Across cultures, there is increasing evidence of considerable sex differences regarding mate choice in humans (Conroy-Beam et al., 2015). While women are typically attracted by men who have power (Fieder and Huber, 2012) or qualities closely related to power, such as money, status, and ambition (Buss, 1989), these are not things that men will typically look for in a woman (Buss, 1989). Many studies indicate that in modern women, there is a negative relationship between socioeconomic status and the number of children (see Huber et al., 2010), while in men the correlation is positive (Fieder and Huber, 2007; Nettle and Pollet, 2008). I have not been able to locate literature indicating that this has been fundamentally different in earlier times or in non-Western cultures. It seems, then, that the presence of autistic traits in women cannot be explained by appealing to natural selection of characteristics that increase the probability of gaining and retaining power.

There is considerable support, however, for the assumption that having a partner with power and resources increases the probability of offspring survival, and that this is why that preference has been selected in the human female (e.g., Wiederman, 1993). Interestingly, there is evidence that female birds and rats engage in more intrasexual competition following experimental elevation of T (Albert et al., 1990; Zysling et al., 2006), and a correlation appears to exist between T level and intrasexual competitiveness in women, so that within an individual, the motivation to compete with other women for mating opportunities appears to increase with salivary T (Hahn et al., 2016).

Elevated androgen levels in women with autism and/or their mothers also seem to play a causative role with regard to autism (see Auyeung and Baron-Cohen, 2013). Findings such as those of Hahn et al. (2016) make it an interesting question whether women high on autistic traits (presumably often reflecting high present and/or prenatal T levels) engage in more intrasexual competition than do typical women. If high T thus increases women’s reproductive success, this could help explain why many women have many autistic traits.

Whether or not women with subclinical autistic traits engage in more intrasexual competition, it should be stressed that the main hypothesis of the present paper is still that autistic traits can help women as well as men acquire and retain power. Furthermore, in women as well as men, T can increase the rate of autistic and power-related behavior, the two often being the same.

## Conclusion

A reviewer pointed out that the paper’s main hypothesis can be put as a strong or a weaker version. It may be that most people with power have subclinical autistic traits that helped them get where they are – or it may be the case that such traits have helped only a subset of the powerful achieve their positions. Two obvious questions are in need of answers, then: (a) Which proportion of powerful people have many autistic traits? (b) Which proportion of powerful people have been helped on their way to the top by any autistic traits they might have? Based on the extant research, the answer to both questions appears to be: Quite a few. But this is an inexact assertion.

Both power and autistic behavior are the subjects of vigorous research, however, which should help us find more specific answers to these empirical questions. Furthermore, if subgroups of powerful people are identified that do and do not have many autistic traits, this leads to questions regarding differences among members of the two groups, the sort of power they wield, how they achieved it, characteristics of their organizations, etc. Meanwhile, several conclusions can be drawn, based on the findings discussed above.

It was postulated that (i) those who gain and sustain power often have many autistic traits, and that (ii) when autistic traits are found in powerful persons, it is often because those traits have been helpful in gaining and sustaining power. These testable claims appear to have considerable empirical support, and they lead to other propositions that are also testable. Among the most important are the following four:

(1) It was predicted that powerful people and those who have many autistic traits are alike in important ways. This claim was found to be supported by the literature. It should be mentioned, though, that much relevant evidence comes from priming experiments, and though biographies as well as more systematic studies of individuals with real power (e.g., Ludwig, 2002) point in the same direction, it would be good to have more stringent studies focusing specifically on the possible existence of autistic traits in people wielding power in the real world.
(2) An attempt was made to establish if a common mechanism exists that could cause the same behavior in powerful and autistic people. This constitutes a relatively strong test of the claim that these traits can reasonably be called autistic, also in the powerful. Interestingly, a common mechanism does appear to exist: In both groups, there is considerable evidence of a causal connection between testosterone and the traits in question.
(3) Relevant findings concur that having power has increased reproductive success in men, but probably not in women. It follows that evolution should thus select for traits contributing to the achievement of power in men. To the extent that such traits are autistic, they will be selected for, and will hence be found more often in men than in women.
(4) One reason why autistic traits are so prevalent, it would seem, is that humans today are disproportionately the descendants of powerful men.

## Author Contributions

The author confirms being the sole contributor of this work and approved it for publication.

## Conflict of Interest Statement

The author declares that the research was conducted in the absence of any commercial or financial relationships that could be construed as a potential conflict of interest.

## Footnotes

1 Football in the British sense, that is, soccer.

2 de Gaulle was a prisoner of war in World War I. Making five attempts to escape, he was put in a maximum security prison and punished with solitary confinement (Haskew, 2011). Russell was imprisoned twice – for making statements “likely to prejudice His Majesty’s relations with the USA” during World War I (Monk, 1996, p. 521), and for civil disobedience in 1961 (Monk, 2000). Mandela was imprisoned for life for his fight against apartheid (Mandela, 1995).
